# First case of isolated vaginal metastasis from breast cancer treated by surgery

**DOI:** 10.1186/1471-2407-12-479

**Published:** 2012-10-17

**Authors:** Filippo Bellati, Innocenza Palaia, Maria Luisa Gasparri, Angela Musella, Pierluigi Benedetti Panici

**Affiliations:** 1 Department of Gynecology and Obstetrics, “Sapienza” University of Rome, Viale Regina Elena 324, 00161, Rome, Italy

**Keywords:** Breast cancer, Radical surgery, Vaginal metastasis

## Abstract

**Background:**

Breast cancer is a leading cause of death in developed countries. This neoplasm frequently relapses at distant sites such as bone, lung, pleura, brain and liver but rarely in the lower female genital tract.

**Case presentation:**

We present the first case of isolated vaginal breast cancer metastasis and its surgical treatment.

**Conclusion:**

This case report focuses on the importance of an accurate genital tract examination as part of regular follow up in breast cancer survivors. Indeed, after this experience we feel that surgery could be considered a valid option for the treatment of an isolated vaginal metastasis.

## Background

Breast cancer is the most frequent neoplasm in women and remains the a leading cause of death in developed countries [1]. The neoplasm frequently relapses at distant sites such as bone, lung, pleura, brain and liver [2]. Disease recurrences rarely occur in organs of the lower female genital tract, such as ovary or to the endometrium [3]. The metastasis to the cervix, as an isolated event, is a rare manifestation, with a variable frequency ranging from 0.8% to 1.7% [4].

Isolated metastasis to the genital tract is explained as a hematogenous spread from the primary site of disease; mostly, it appears as a concomitant involvement of a broadly disseminated disease [5].

Only two cases of vaginal metastasis from breast cancer have been so far reported in literature [6,7]; both of them are presented as part of a systemic disease involving also endometrium and ovaries.

We present the first case of *isolated* vaginal metastasis, secondary to an unilateral lobular breast cancer, recurring four years after complete clinical remission.

## Case presentation

In February 2008, a 54-year-old woman, with a history of lobular breast cancer T2N1M0, was referred to our Department for vaginal bleeding. In march 2005 she was submitted to left superior quadrantectomy plus axillary lymphadenectomy followed by adjuvant chemotherapy, radiotherapy, and hormonal treatment for 5 years. After completion of adjuvant chemotherapy and radiotherapy, the patient was followed up regularly every 3–6 months by the mean of clinical examination, imaging and laboratory exams. Gynecological examination was performed every year, with normal findings.

At admission she was in good general condition, only complaining an un-painful vaginal bleeding. She was still under hormonal treatment.

**Figure 1:**
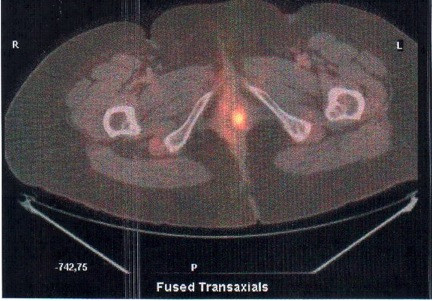
Isolated lesion to left vaginal wall at Total Body CT-PET scan.

The recto-vaginal examination, revealed a 4 cm solid lesion sited at level of the left upper third of the vagina, which involved the full thickness of the vaginal wall and the obturator fossa. The lesion did not involve the cervix and the trans-vaginal ultrasound did not reveal endometrial or adnexal abnormalities. However a hysteroscopy with endometrial biopsy was performed to exclude endometrial involvement, which was negative. Tumor markers were all within normal range. A Total Body CT-PET scan was performed which showed an isolated iper-captation on the left vaginal wall (Figure 1). A vaginal biopsy confirmed the breast cancer metastatic nature of the nodule.

**Figure 2:**
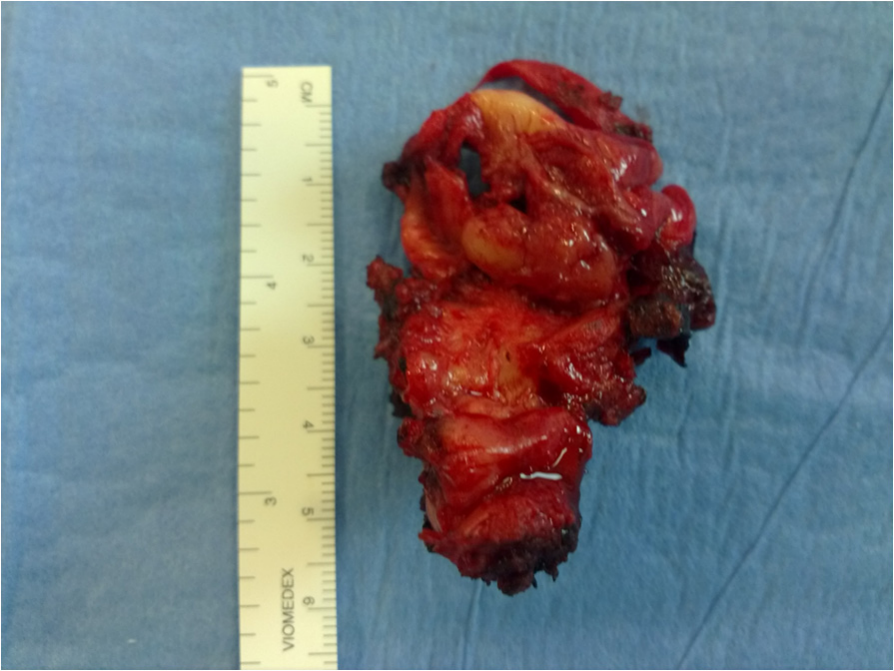
Surgical specimen of vaginal lesion.

After careful counseling on the various therapeutic options, it was decided with the patient to treat the vaginal relapse with surgery. She was subjected to a modified vaginectomy [8] with complete resection of the mass (Figure 2) together with a large amount of lympho-fatty tissue around the lesion. Pathology showed a metastatic poorly differentiated lobular breast cancer. Immunohystochemistry showed expression of estrogen and progesterone receptors in 90% and none of neoplastic cells respectively and negative C-Erb-2 expression consistently with the primary tumor. Resection margins were negative.

The patient was subjected to adjuvant chemotherapy with Pegilated Liposomal Doxorubicin 40 mg/mq for 6 courses.

After 14 months of negative follow up, we decided to perform an elective completion of surgery with prophylactic aims. A total hysterectomy plus bilateral salpingo-oophorectomy was performed. Surprising, Hystological Examination documented. Again micrometastasis from lobular breast cancer in the contest both of the uterus and ovaries. No adjuvant treatment was proposed.

Twelve months later, the patient is still free from disease.

## Discussion and conclusion

Metastases to the female genital tract from breast cancer are unusual.

Defining “breast unusual metastasis” as rare systemic failure with a frequency of <1% [3].

Lobular histotype seems to metastasize to the genital tract more frequently than ductal tumors [9], probably for an hematogenous propagation.

Few cases are reported in literature describing isolated metastasis to the cervix [4-8,10]. To our knowledge, this is the first case of an *isolated* vaginal relapse from breast cancer treated by surgery.

Data regarding vaginal metastasis behavior and treatment are scarce. Most of the cases are treated by radiotherapy [6,7]. In our opinion, surgery is a valid alternative to radiotherapy , and the absence of relevant side effects or complication and the current status of our patient corroborate this strategy.

Furthermore, this case report suggests that cancer survivors should be subjected to a more thorough gynecologic examination. Although a baseline gynecologic assessment is recommended prior to administration of the cancer risk reduction agents (Tamoxifen), and follow up gynecologic assessment should be performed at each visit, as assess by NCCN Guidelines, currently gynecological counseling is not universally part of breast cancer follow up workup.

Globally our experience highlights that breast cancer survivors deserve a periodic gynecological assessment as part of their regular follow up. Also, we can assume that recurrent disease would have been undetected if deeper evaluations had not be carried out and that when it occurs surgery could be a rationale choice of treatment.

In conclusion, in our opinion, in case of recurrence in female genital tract an expert Gynecologic Oncology Surgeon should be consulted.

## Consent

Written informed consent was obtained from the patient for publication of this Case report and any accompanying images. A copy of the written consent is available for review by the Series Editor of this journal.

## Competing interests

The authors declare that they have no competing interests.

## Authors’ contributions

FB drafted the manuscript. IP conceived of the study. MLG participated in the design of the study. AM participated in the sequence alignment. PBP manages the patient and coordinates the design of the study. All Authors read and approved the final manuscript.

## Pre-publication history

The pre-publication history for this paper can be accessed here:

http://www.biomedcentral.com/1471-2407/12/479/prepub
